# Closed Or Open after Source Control Laparotomy for Severe Complicated Intra-Abdominal Sepsis (the COOL trial): study protocol for a randomized controlled trial

**DOI:** 10.1186/s13017-018-0183-4

**Published:** 2018-06-22

**Authors:** Andrew W. Kirkpatrick, Federico Coccolini, Luca Ansaloni, Derek J. Roberts, Matti Tolonen, Jessica L. McKee, Ari Leppaniemi, Peter Faris, Christopher J. Doig, Fausto Catena, Timothy Fabian, Craig N. Jenne, Osvaldo Chiara, Paul Kubes, Braden Manns, Yoram Kluger, Gustavo P. Fraga, Bruno M. Pereira, Jose J. Diaz, Michael Sugrue, Ernest E. Moore, Jianan Ren, Chad G. Ball, Raul Coimbra, Zsolt J. Balogh, Fikri M. Abu-Zidan, Elijah Dixon, Walter Biffl, Anthony MacLean, Ian Ball, John Drover, Paul B. McBeth, Juan G. Posadas-Calleja, Neil G. Parry, Salomone Di Saverio, Carlos A. Ordonez, Jimmy Xiao, Massimo Sartelli, for The Closed Or Open after Laparotomy (COOL) after Source Control for Severe Complicated Intra-Abdominal Sepsis Investigators

**Affiliations:** 1 Department of Surgery, University of Calgary, Calgary, Alberta Canada; 2 Department of Critical Care Medicine, University of Calgary, Calgary, Alberta Canada; 3 The Trauma Program, University of Calgary, Calgary, Alberta Canada; 4 General, Emergency and Trauma Surgery Department, Bufalini Hospital, Cesena, Italy; 5 Unit of General and Emergency Surgery, Bufalini Hospital of Cesena, Cesena, Italy; 6 Department of Abdominal Surgery, Abdominal Center, University of Helsinki and Helsinki University Central Hospital, Helsinki, Finland; 7 Regional Trauma Services, Foothills Medical Centre, Calgary, Alberta Canada; 8 Research Facilitation Analytics (DIMR), University of Calgary, Calgary, Alberta Canada; 9 Department of Community Health Sciences, Cumming School of Medicine, University of Calgary, Calgary, Alberta Canada; 10 Emergency Surgery Department, Parma University Hospital, Parma, Italy; 11 Surgery, University of Tennessee Health Sciences Center Memphis, Memphis, TN USA; 12 General Surgery and Trauma Team Niguarda Hospital Milano, Milan, Italy; 13 Calvin, Phoebe and Joan Snyder Institute for Chronic Diseases, University of Calgary, Calgary, Alberta Canada; 14 Department of Physiology, Cumming School of Medicine, University of Calgary, Calgary, Alberta Canada; 15 Department of Pharmacology, Cumming School of Medicine, University of Calgary, Calgary, Alberta Canada; 16 Department of Medicine, University of Calgary, Calgary, Alberta Canada; 17 Libin Cardiovascular Institute and O’Brien Institute of Public Health, University of Calgary, Calgary, Alberta Canada; 18 Rambam Health Care Campus, Haifa, Israel; 19 Division of Trauma Surgery, University of Campinas, Campinas, SP Brazil; 20 Department of Surgery, R Adams Cowley Shock Trauma Center, University of Maryland School on Medicine, Baltimore, MD USA; 21 Donegal Clinical Research Academy, Letterkenny University Hospital, Donegal, Ireland; 22 Trauma and Critical Care Research, University of Colorado, Denver, CO USA; 23 Department of Surgery, Jinling Hospital, Medical School of Nanjing University, Nanjing, China; 24 General, Acute Care, and Hepatobiliary Surgery, and Regional Trauma Services, University of Calgary, Calgary, Alberta Canada; 25 Riverside University Health System Medical Center, Loma Linda, CA USA; 26 Department of Surgery, Loma Linda University School of Medicine, Loma Linda, CA USA; 27 John Hunter Hospital and Hunter New England Health District, Newcastle, NSW Australia; 28 Surgery and Traumatology, University of Newcastle, Newcastle, NSW Australia; 29 Department of Surgery, College of Medicine and Health Sciences, UAE University, Al-Ain, United Arab Emirates; 30 Surgical Oncology, University of Calgary, Calgary, Alberta Canada; 31 City Wide Section of General Surgery, University of Calgary, Calgary, Alberta Canada; 32 Scripps Memorial Hospital La Jolla, La Jolla, California USA; 33 Department of Medicine, Western University, London, Ontario Canada; 34 Department of Epidemiology and Biostatistics, Western University, London, Ontario Canada; 35 Department of Critical Care Medicine, Queen’s University, Kingston, Ontario Canada; 36 Department of Surgery, Queen’s University, Kingston, Ontario Canada; 37 Department of Surgery, Western University, Victoria Hospital, London Health Sciences Centre, London, Ontario Canada; 38 Department of Critical Care, Western University, Victoria Hospital, London Health Sciences Centre, London, Ontario Canada; 39 Addenbrooke’s Hospital, Cambridge University Hospitals NHS Foundation Trust, Cambridge, UK; 40 Department of Surgery, Fundación Valle del Lili and Universidad Del Valle, Cali, Colombia; 41 Department of Surgery, Macerata Hospital, Macerata, Italy

**Keywords:** Intra-peritoneal sepsis, Septic shock, Peritonitis, Open-abdomen, Multiple organ dysfunction, Laparotomy, Randomized trial, Bio-mediators

## Abstract

**Background:**

Severe complicated intra-abdominal sepsis (SCIAS) has an increasing incidence with mortality rates over 80% in some settings. Mortality typically results from disruption of the gastrointestinal tract, progressive and self-perpetuating bio-mediator generation, systemic inflammation, and multiple organ failure. Principles of treatment include early antibiotic administration and operative source control. A further therapeutic option may be open abdomen (OA) management with active negative peritoneal pressure therapy (ANPPT) to remove inflammatory ascites and ameliorate the systemic damage from SCIAS. Although there is now a biologic rationale for such an intervention as well as non-standardized and erratic clinical utilization, this remains a novel therapy with potential side effects and clinical equipoise.

**Methods:**

The Closed Or Open after Laparotomy (COOL) study will constitute a prospective randomized controlled trial that will randomly allocate eligible surgical patients intra-operatively to either formal closure of the fascia or use of the OA with application of an ANPTT dressing. Patients will be eligible if they have free uncontained intra-peritoneal contamination and physiologic derangements exemplified by septic shock OR a Predisposition-Infection-Response-Organ Dysfunction Score ≥ 3 or a World-Society-of-Emergency-Surgery-Sepsis-Severity-Score ≥ 8. The primary outcome will be 90-day survival. Secondary outcomes will be logistical, physiologic, safety, bio-mediators, microbiological, quality of life, and health-care costs. Secondary outcomes will include days free of ICU, ventilation, renal replacement therapy, and hospital at 30 days from the index laparotomy. Physiologic secondary outcomes will include changes in intensive care unit illness severity scores after laparotomy. Bio-mediator outcomes for participating centers will involve measurement of interleukin (IL)-6 and IL-10, procalcitonin, activated protein C (APC), high-mobility group box protein-1, complement factors, and mitochondrial DNA. Economic outcomes will comprise standard costing for utilization of health-care resources.

**Discussion:**

Although facial closure after SCIAS is considered the current standard of care, many reports are suggesting that OA management may improve outcomes in these patients. This trial will be powered to demonstrate a mortality difference in this highly lethal and morbid condition to ensure critically ill patients are receiving the best care possible and not being harmed by inappropriate therapies based on opinion only.

**Trial registration:**

ClinicalTrials.gov, NCT03163095.

## Background

Sepsis is an ever-increasing cause of death worldwide [1, 2], with a current incidence that is estimated at between 18 to 31 million cases worldwide per year [2–6]. Mortality approaches 30–40% when shock is present [7–9], although this may be 80% in the developing world [1]. Intra-abdominal sepsis (IAS) constitutes the second most common form of sepsis, which may be particularly severe because of the unique anatomic, physiologic, and microbiologic characteristics of the abdominal cavity and its contained hollow viscera [10]. Thus, it has been reported that hospital mortality is highest for patients who have intra-abdominal infection secondary to ischemic bowel or disseminated infection [11].

Severe complicated intra-abdominal sepsis (SCIAS) encompasses the most challenging situation physicians and surgeons encounter. IAS is defined as severe when associated with organ dysfunction [8, 12–14] and as complicated when the inflammation or contamination spreads beyond a single organ, causing either localized or diffuse peritonitis [12, 15]. SCIAS, typically resulting from secondary peritonitis, may be distinguished from other causes of severe sepsis through a requirement for surgical abdominal exploration to surgically address the disruption in the gastrointestinal (GI) tract.

Patients with SCIAS require early hemodynamic support, source control, and antimicrobial therapy [15]. However, despite advances in diagnosis, surgery, and antimicrobial therapy, mortality rates associated with complicated intra-abdominal infections and IAS remain exceedingly high [14]. Even with prompt appropriate therapy, SCIAS may progress to septic shock and multiple organ dysfunction, largely because of peritoneal and systemic inflammation. There is great variability in the human immune response to an infectious focus, and some individuals greatly overreact to an inciting infection and produce a massive bio-mediator storm that propagates multi-system organ failure and death whereas other individuals have little or no response to the same stimuli. The failure to obtain adequate source control is often the driving cause of SCIAS and has been identified as an independent predictor of mortality in those with this condition [16].

In patients with SCIAS, relaparotomy is often necessary to eliminate persistent peritonitis or new infectious foci [17–19]. Differentiating “failed source control” [20, 21] from a self-propagating bio-mediator storm is often difficult or impossible without abdominal re-exploration (relaparotomy). In those randomized to expectant management after laparotomy for intra-abdominal sepsis, 42% still required relaparotomy for suspected or proven persistent peritonitis in a large Dutch multicenter randomized controlled trial (RCT) conducted by van Ruler and colleagues [17]. Interestingly, in this study, 31% of these patients had a negative relaparotomy. The results of this seminal study, however, largely concluded a previously long-standing debate concerning two surgical approaches to ensuring source control in the peritoneal cavity that of “laparotomy on demand” (LOD) versus “planned re-laparotomy” (PRL) [17, 22, 23].

In a PRL strategy, re-laparotomy with fascial closure is routinely performed every 36–48 h in order to inspect, drain, lavage, and apply any other required source control for the abdominal cavity until the intra-operative findings are negative for peritonitis [17]. LOD offers repeat laparotomy only in those patients in whom the lack of clinical improvement or even clinical deterioration suggests that ongoing peritonitis results from either persistent peritonitis or a new infectious focus [17]. The relative merits of either approach were widely debated for years, until the conclusion of the above RCT [17]. Although this trial noted no difference in mortality between the two methods, the LOD strategy reduced direct medical costs by 23% [17].

The equivalence in outcomes, coupled with an apparent cost savings, resulted in the generation of consensus guidelines recommending that LOD after laparotomy for SCIAS be adopted as the standard of care [24]. Upon critical review, however, the mortality in this RCT of severe secondary peritonitis well illustrates the devastating nature of this disease, noting the associated mortality of approximately one third of all enrolled patients regardless of treatment allocation. No matter which cohort is considered, this dismal outcome demands for the design of alternative approaches to manage SCIAS in an attempt to save more lives.

At present, pharmacologic approaches are not the answer. Despite the substantial improvement in supportive critical care that has occurred over time, there has not been seminal advances in addressing the central dysregulated inflammation ultimately causing the organ damage that kills or maims patients with severe sepsis. Attempts to derive pharmacologic therapies for combating post-infective inflammation have proved to be incredibly expensive and frustrating, with hundreds of failed anti-mediator trials having been conducted without evidence of significantly improved patient outcomes [25, 26].

A critical nuance to consider in understanding surgical source control is that the van Ruler RCT did not utilize a contemporary “open abdomen” (OA) approach in either arms and that the abdominal fascia was formally closed in both. Increasingly, the OA is being recommended as an attractive option to provide better control of intraperitoneal contamination. Now, this approach is perceived to be a safer option than in previous decades due to the development of advanced temporary abdominal closure (TAC) devices, that offer greater safety in protecting the viscera, and their potentially profound benefits in ameliorating the propagation of inflammatory bio-mediators in SCIAS [27–29].

The use of the OA for non-trauma general surgery is increasingly being reported in uncontrolled series as a potentially beneficial option for patients with SCIAS [12, 20, 21, 30–32]. The use of the OA in severe sepsis may offer early identification and increased drainage of any residual infection, control any persistent source of infection, more effectively remove bio-mediator-rich peritoneal fluid, provide prophylaxis against development of the abdominal compartment syndrome, and allow for the safe deferral of gastrointestinal anastomoses and a safer exit at the index operation [12]. Compared to trauma patients, however, patients undergoing OA management for intra-abdominal sepsis have a greater risk of OA complications, including enteroatmospheric fistula (EAF) and intra-abdominal abscess formation, and a lower rate of primary fascial closure (i.e., fascia-to-fascia closure within the index hospitalization) [12, 13, 33–35].

Although case series reporting the use of the OA after non-trauma laparotomies have been reported, there are no other contemporary randomized studies to address this critical issue. There has only been one other RCT conducted prior to 2006 that randomized patients to a closed or open strategy, but the techniques of OA management used were inadequate as management of the OA has undergone dramatic improvements in technology and technique in recent years. Robledo and colleagues randomized patients with severe secondary peritonitis to an open or closed strategy after laparotomy, using a non-absorbable polypropylene (Marlex™) mesh in a interposed position between the open fascia, thus exposing the underlying bowel to great risk of enterocutaneous or enteroatmospheric fistula formation [36]. The study was stopped at the first interim analysis. Although the mortality difference between the two groups did not reach statistical significance, the risk of death was higher with the OA, interposed non-absorbable polypropylene mesh strategy [36].

Although RCT data comparing techniques are needed, meta-analyses conducted by our group [37] and the Amsterdam group [34] have concluded that negative pressure wound therapy (NPWT) treatment appears to potentially be the safest and most effective OA management technique currently available. Newer commercial active negative pressure peritoneal therapy (ANPPT) systems now available for OA may reduce the risks of enterocutaneous fistula and facilitate enhanced delivery of negative peritoneal pressure to the peritoneal cavity [24, 37, 38]. Animal studies [39] and in silica modeling of these animal studies [40] have shown that ANNPT provides a greater degree of negative pressure throughout the peritoneum, which may reduce plasma bio-mediator levels when compared to more passive peritoneal drainage. Systemic inflammation (TNF-α, IL-1β, IL-6) in one study was significantly reduced in the ANPPT group and was associated with significant improvement in intestine, lung, kidney, and liver histopathology [39]. Although the mortality rate in the NPPT was 17 versus 50% in the control group, this difference was not statistically significant (*p* = 0.19), likely due to the smaller numbers. A larger prospective, but non-randomized, multi-center cohort study in critically ill/injured patients requiring an OA enrolled 280 patients from 20 sites, in whom 168 underwent at least 48 h of consistent OA therapy [28], and compared ANPPT and a second type of TAC that provides potentially less efficient peritoneal negative pressure. Although bio-mediator levels were not measured in this trial, the 30-day all-cause mortality rate was 14% in those treated with ANPPT and 50% in those with the less efficient negative pressure TAC [28].

Our research group has conducted the only prospective randomized controlled trial addressing this question, the Intraperitoneal Vacuum Trial [27]. This RCT, conducted in Calgary, Alberta, enrolled 45 out of 63 potentially eligible patients over a 15-month period between September 2011 and December 2012. Patients were enrolled in the operating room after an attending surgeon made the decision that an abbreviated laparotomy was required in critically ill/injured patients. In addition to numerous physiological variables, bio-mediator levels were measured every 24 h in the initial post-laparotomy phase of critical care [27, 41]. Although standard systemic bio-mediator levels were not statistically different nor were peritoneal fluid drainage, the 90-day survival rate was improved in the ANPPT group (hazard ratio, 0.32; 95% confidence interval, 0.11–0.93; *P* = 0.04) [27]. A valid critique of this trial was that despite the fact that all patients were deemed to need OA therapy by the attending surgeon, there was still a rather heterogeneous mix of trauma and non-trauma patients [27]. Thus, although unexplained, significantly improved survival with ANPPT does warrant further exploration as a potential treatment in patients affected by severe SCIAS. We therefore believe that the global clinical equipoise as to whether the abdomen should be left open or closed after laparotomy in patients with SCIAS warrants a carefully conducted multicenter RCT [30, 42].

## Methods/design

### Objective/aims

The aim of the study is to test the null hypothesis that there will be no difference in survival when an OA management strategy administering ANPPT is utilized compared to a primary fascial closure strategy in patients with SCIAS. The study will be designed as a prospective, single-blinded, multi-center, international RCT. A SPIRIT diagram overview of the trial is presented in Table 1. The complete protocol is available at https://coolstudy.ca/.

**Table 1:**
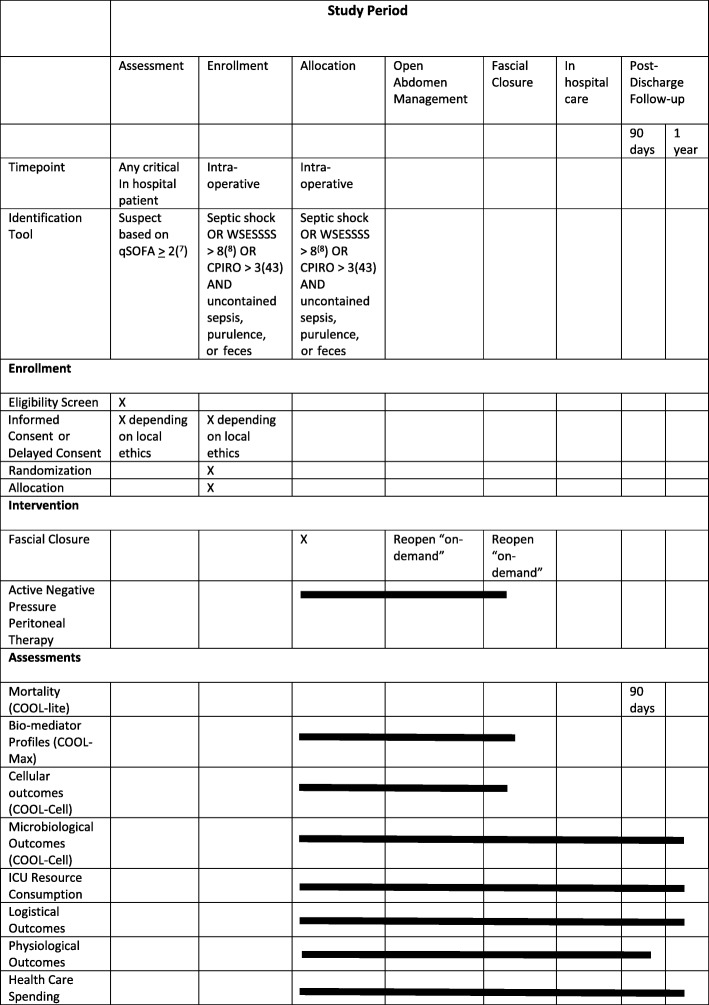
SPIRIT diagram describing schedule of enrolment, interventions, and assessments [43]

### Setting

The study will be conducted in operating rooms around the world where critically ill patients with SCIAS undergo source control laparotomy. The lead study center will be the Foothills Medical Centre, a Quaternary Care Academic Medical Centre located in Calgary, Alberta, Canada. Other recruiting sites will be located in developed countries around the world and will include academic and community hospitals with the resources necessary to participate in the trial and care for patients with SCIAS during the entire clinical follow-up period.

### Inclusion/exclusion criteria

**Fig. 1:**
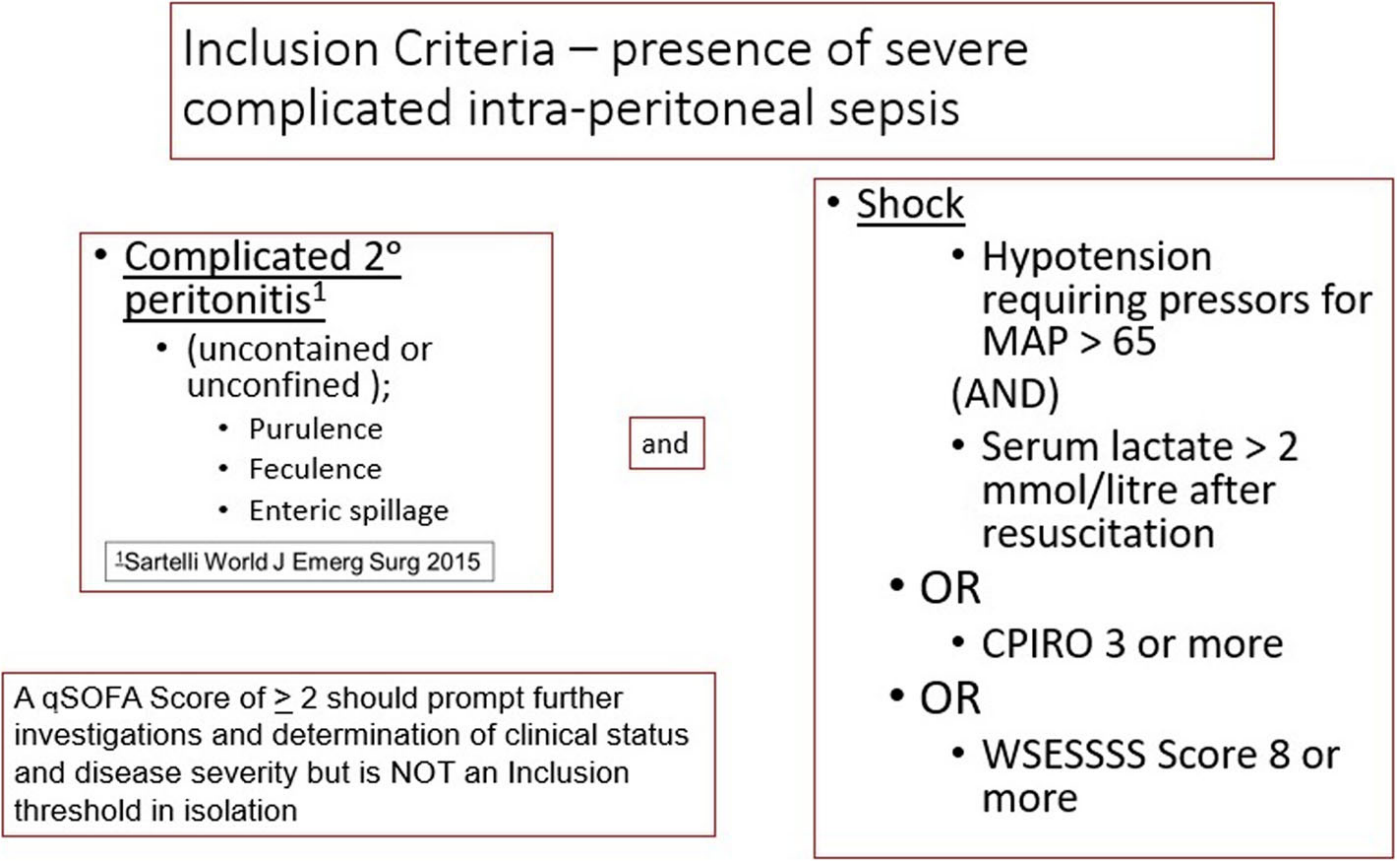
Inclusion criteria for COOL

Potential patients will first be identified in the emergency departments, inpatient ward, and critical care units of the participating centers. Eligibility will then be confirmed in the operating room during the conduct and near completion of a laparotomy for source control. Patients will be eligible for inclusion if they have SCIAS, as operationally defined by the COOL trial (Fig. 1).

The inclusion criteria are conceptually a two-part assessment to ascertain if patients clearly fulfill the definition of both severe and complicated IAS while undergoing source control laparotomy. Thus, during the laparotomy, it will become apparent to the operating surgical team that the peritonitis is complicated, which will be reproducibly demonstrated by uncontained or unconfined purulent, feculent, or enteric spillage. In addition to being complicated, the inclusion criteria require that patients have severe IAS. For the purpose of the COOL study, severe will be defined by any of septic shock as defined by Sepsis 3 Consensus Guidelines [7], a World Society of Emergency Surgery Sepsis Severity Score ≥ 8 [8], or a Calgary Predisposition-Infection-Response-Organ Dysfunction Score ≥ 3 [43]. An elaborated explanation of the thought processes and identification attributes of these criteria modeled on a trial population of SCIAS patients was previously published by the COOL investigators [44].

The qSOFA score was developed as a simple clinical tool to identify patients with suspected infection who were likely to have poor outcomes that did not rely on laboratory testing [7]. However, during modeling, the qSOFA actually had the lowest AUC of the systems formally tested and a lower identification rates than the other systems selected. This was consistent with other opinions that the qSOFA might not be a sensitive enough tool [45–48]. Therefore, although the qSOFA score ≥ 2 is not an inclusion criterion, its ease of use on the ward is attractive to serve as a flag for patients with IAS in whom caregivers should assess further whether critical features of SCIAS are present.

The exclusion criteria for COOL include (a) pregnancy, (b) perceived physical inability to physically close the fascia primarily without undue tension or concerns for inducing severe IAH/ACS, (c) intra-operatively determined absolute or imperative requirement for “damage control” laparotomy including intra-peritoneal packing or non-anatomic post-surgical anatomy (i.e., surgically placed permanent packing or bowel that the operating surgeon believes must be left in discontinuity after resection), (d) the patient is expected to die shortly after operation because of their condition in the operating room and there is no intention of providing ongoing care (i.e., the treating team wishes to close the abdomen to leave the operating room with the sole intention of withdrawing aggressive measures and providing only “comfort care” in the ICU; an example of where this could occur would be complete transmural midgut ischemia/necrosis), (e) laparoscopic surgery (no laparotomy), (f) pancreatitis as the source of peritonitis, (g) acute superior mesenteric artery occlusion as the primary pathology, (h) co-enrollment in another investigational study, (i) peritoneal carcinomatosis, (j) traumatic injury within 24 h of the development of SCIAS, (k) age < 18, or (l) uncontrolled bleeding. It will be important for surgeons considering recruiting a patient to recognize before enrolling and randomizing a patient that fascial closure is not possible, as recognizing this after allocation to closure will constitute a protocol violation.

In current practice, it is likely that the most common reason for non-eligibility will be a surgeon-based decision to resect a hollow viscus and due to the perceived critical nature of the patient decision not to re-anastomose the bowel but to instead perform damage control and return the bowel ends into the peritoneal cavity without a diverting stoma. As this is an absolute indication for a future re-operation, these patients will be ineligible for randomization. Although some authors are critical of this practice [49], others recognize or even recommend this approach [20, 30, 42, 49–51]. This group of patients will be expected to constitute a significant and important population of very sick patients who although non-randomized and excluded constitute a “defacto third arm” requiring follow-up and outcome description.

### Randomization

Treatment arm allocation will be randomly allocated from a central, password protected, randomization website (https://coolstudy.ca/). This will require an operating room with access to the Internet, although accessing the enrollment site needs not to be conducted by an actual member of the surgical team. This site will be freely open to the public; however, the ability to enroll a patient can only be accessed with a password by any member of the surgical/anesthesia/critical care medicine/nursing team, thus freeing the senior surgeon to concentrate on care. When an appropriate patient is recognized, the research website will be accessed, simple identifiers of the patient will be entered, and treatment allocation (CLOSED with fascial closure or OPEN with an ANPPT TAC being applied) associated with this entry will be generated. To ensure close balance of the numbers in each of the two treatment groups, permuted block randomization by site will be used. If the operating team is uncertain regarding the potential stratified severity according to either the WSESSSS or CPIRO methods, online decision support software will greatly simplify these calculations regarding any potential enrollment.

### Primary closure—CLOSED allocation

This strategy will consist of primary closure of the fascia with placement of a closed-suction intra-peritoneal drain (such as a Jackson-Pratt or Blake drain) to allow drainage of intra-peritoneal fluid for both clinical reasons and to facilitate intra-peritoneal fluid testing. Closure of the skin and the method for preventing surgical site infections will be left to the discretion of the attending surgeon. There will be no formal requirement for relaparotomy. Post-operative diagnostic imaging and all other aspects of post-operative care shall be at the discretion of the treating critical care/surgical teams. Any decision to perform a relaparotomy will be at the discretion of the treating critical care/surgical teams, and in no way mandated by this study, although this will constitute a study outcome. If at any subsequent laparotomy, the attending and responsible surgeon selects an open abdominal strategy as being in the patient’s best interest, this will be permitted and the outcomes will be analyzed considering the original intention to treat allocation at enrollment. Any application of any wound suction or negative pressure device to the soft tissue above the closed fascia will be permitted but will not change the understanding that the fascia has been formally closed and this is a patient with a closed abdomen.

### Open abdomen with active negative pressure peritoneal therapy—OPEN allocation

The time that the TAC dressing will be left in place will be left to the discretion of the attending surgeon, but typical practice guidelines mandate either formal abdominal closure or dressing changes at 24–72 h from placement if it is not perceived that formal abdominal closure can be completed [52] . For both arms of the trial, it will be expected that attending surgeons are involved in either the direct supervision or inter-operative participation with either facial closure or temporary abdominal closure in order to be an acceptable participating center. The trial is considered pragmatic in allowing a variety of techniques as long as ANPPT is being administered to an open peritoneal cavity defined by the fascia not being formally closed and that all four intra-peritoneal quadrants have been washed until macroscopically clean [24]. Thus, any manner of mechanical traction devices [53, 54], or potential instillation therapies [55], will be permitted adjuncts as long as the primary requirement for an open fascia with ANPPT is met.

### Clinical management protocols

After enrolment and allocation of abdominal compartment management in the operating theater, all care will be at the complete discretion of the clinical teams. If the treating physicians decide that a CLOSED abdomen requires re-opening, participation in the study will not influence this decision in any way. However, this will be an outcome and the case will still be analyzed as CLOSED in the primary intention to treat analysis. The timing of re-operation for an OPEN abdomen will be at the discretion of the treating physicians and not dictated by this trial although the planned secondary determinations of bio-mediator profiles will be analyzed on a per-protocol basis only for patients that had at least 24 h of continuous OPEN or CLOSED management as allocated.

### Outcomes

The primary outcome will be survival at 90 days. Secondary outcomes will be considered logistical, physiologic, as well as outcomes that will enable an economic analysis (a measure of utility and health-care costs). Logistical outcomes will include days free of (DFO) ICU, ventilation, renal replacement therapy, and hospital at 30 days from the index laparotomy. The physiological secondary outcomes will include change in APACHE II, SOFA, and ARDS scores after laparotomy. The COOL study inclusion criteria concerning intra-peritoneal contamination will be recorded, and the index source control laparotomy and every subsequent laparotomy will be graded according to the OA classification system from 2013 World Society of Abdominal Compartment Syndrome (WSACS) grading scale for OA [24, 56, 57]. Surgical complications occurring after the index laparotomy will be graded according to Clavien-Dindo (grade I = any deviation from normal postoperative course, including wound infections opened at the bedside but not treated with antibiotics; grade II = requiring pharmacological treatment, e.g., antibiotic treatment, blood transfusion, or parenteral nutrition; grade IIIa = requiring surgical, endoscopic, or radiologic intervention without general anesthesia and grade IIIb = under general anesthesia; grade IVa = life-threatening complication requiring IC/ICU management with single organ dysfunction and grade IVb = with multiorgan dysfunction; grade V = death of patient) [58, 59].

Bio-mediator outcomes for centers participating in COOL-Max will consist of the measurement of IL-6 and 10, procalcitonin, activated protein C (APC), high-mobility group box protein 1, C3a, C5a, and mitochondrial DNA. To enable a full economic analysis, we will measure 1-year health-care costs using administrative data and assess overall quality of life at 90 days and 1 year using the Euroqol EQ-5D standardized survey with translations into participants first language where necessary. An overview of the study outcomes is presented in Table 2.

**Table 2:** Overview of study outcomes

	Indicator	Timeline
Primary outcome	Mortality	90 days
Secondary outcomes		
Logistical	Days free of ICU	30 days
	Days free of ventilation	30 days
	Days free of RRT^a^	30 days
	Days free of hospital	30 days
Physiological	APACHE II^b^ scores	Up to 30 days^c^
	SOFA^d^ scores	Up to 30 days^c^
	Pa0_2_/Fi0_2_^e^ ratios	Up to 30 days^c^
	ARDS^f^ scores	Up to 30 days^c^
Safety	Enterocutaneous fistula	30 days
	ACS^g^ and/or severe IAH^h^	30 days
	Intra-abdominal abscess	30 days
Biological	Il-6	Up to 30 days^i^
	IL-10	Up to 30 days^i^
	Procalcitonin	Up to 30 days^i^
	Activated protein C	Up to 30 days^i^
	High mobility group box protein 1	Up to 30 days^i^
	Mitochondrial DNA	Up to 30 days^i^
	C3a and C5a	Up to 30 days^i^
Microbiological	Intra-abdominal	Up to 30 days^j^
	Microbiological cultures	
Mass cytometry	Intra-peritoneal inflammatory cells	Up to 30 days^k^
Economic	Micro-costed resource consumption	1 year
Quality of life	Euroqol EQ-5D-5L	90 days and 1 year
	SF-36	90 days and 1 year

^a^*RRT* renal replacement therapy

^b^Acute Physiology and Chronic Health Evaluation Score

^c^Measured daily using the worst value of that day

^d^*SOFA* Sequential Organ Failure Assessment

^e^*Pa0*_*2*_*/Fi0*_*2*_ partial pressure of oxygen over inspired fraction of oxygen

^f^*ARDS* Acute Respiratory Distress Syndrome

^g^*ACS* Abdominal Compartment Syndrome

^h^*IAH* intraabdominal hypertension

^i^Measured as per Table 3

^j^Measured as clinically indicated by the treating team

^k^Measured on intra-peritoneal fluid obtained in Calgary

### COOL: COOL-Lite, COOL-Max, COOL-Mic, COOL-Cells, and COOL-Costs

The study will be powered to detect a mortality difference between the two allocated therapies which will be the most valuable deliverable of the study. Thus, any dedicated site can participate if they are committed to recruit and randomize patients with SCIAS fulfilling the eligibility criteria during source control laparotomies. Contributing towards this main outcome will require only collection of the clinical outcome data and is known as “COOL-Lite” participation in the study. Interested sites that have the capability to collect and store biological samples however will also be encouraged to participate in the “COOL-Max,” in which samples of both serum and peritoneal fluid will be collected to understand the evolution of bio-mediators in the course of SCIAS and to determine differential effects of open or closed abdominal therapy on these profiles. Participation in COOL-Max will involve collection of bio-mediator samples for a prior analysis as stipulated in Table 3. Participating in the COOL-Max effort will however equate to the collection of serum and peritoneal fluid which will be stored in a frozen state permitting other secondary and tertiary analyses correlated to COOL clinical outcomes in response to questions and avenues for scientific exploration as a result of this study. Other concurrent science that may be conducted in accordance with the main COOL trial will a COOL microbiology (COOL-Mic) arm that will consider the microbiology of secondary peritonitis in the OA arm of COOL-Lite and to follow the subsequent modifications in microbiologic flora including patients in the CLOSED arm who require reoperation; an analysis of the behavior of innate intra-peritoneal cellular defense mechanisms will be undertaken (COOL-Cells). Analysis of bio-mediator profile kinetics/dynamics will be on a “per-protocol basis” with per-protocol compliance requiring the delivery of at least 24 h of allocated treatment before any potential cross-over occurred regarding CLOSED or OPEN allocation.

**Table 3:** Summarized bio-mediator samples for COOL-MAX centers

Will be drawn from both the serum and peritoneal fluid	
Timing of sample collection	
- Enrollment in the OR	
- 6 h post enrollment	
- 12 h post enrollment	
- 18 h post enrollment	
- 24 h post enrollment	
- 36 h post enrollment	
- 48 h post enrollment	
- 72 h post enrollment	
- 168 h (7 days) post enrollment	
- 336 h (14 days) post enrollment	
- 720 h (30 days) post enrollment	

For those patients recruited in Calgary (and potentially other geographically close sites in Alberta), mass cytometry specimens will be collected from the peritoneal fluid when possible (COOL-Cells). Mass cytometry is a mass spectrometry technique based on inductively coupled plasma mass spectrometry and time of flight mass spectrometry used for the determination of the properties of cells (cytometry). In this approach, antibodies are conjugated with isotopically pure elements, and these antibodies are used to label cellular proteins. Cells are nebulized and sent through an argon plasma, which ionizes the metal-conjugated antibodies. The metal signals are then analyzed by a time-of-flight mass spectrometer. The approach overcomes limitations of spectral overlap in flow cytometry by utilizing discrete isotopes as a reporter system instead of traditional fluorophores which have broad emission spectra. These sub-studies are not the focus of the present document, and COOL itself will be powered for survival considering the basic COOL-Lite protocol.

Finally, in COOL-Costs, we will use information on survival (which can be extrapolated to life expectancy), quality of life, and health-care costs to conduct a full economic evaluation. Overall quality of life will be assessed using the SF-36 and Euroqol EQ-5D-5L at 90 days and 1 year post-enrollment in survivors, either by paper or by phone, which has been used extensively in ICU survivors. The potential resource implications of the intervention include the cost of the strategy itself (which will be assessed using a microcosting approach) and include any implications on surgeries performed, ICU and hospital stay, any costs associated with adverse events of the treatment strategy, the costs associated with renal replacement therapy use after ICU discharge, and subsequent hospital readmissions. To measure these impacts, we will assess hospital and ICU length of stay, the number of surgeries performed (and types), physician interactions, and subsequent use of RRT for all patients within the trial, with valuation of all costs being based on all patients enrolled within Alberta, and other participating sites able to provide administrative and microcosting data. Microcosting data is available for all hospitalizations within sites in Alberta, which will enable an accurate evaluation of ICU costs separate from hospital ward costs—critical to this analysis. Additional health administrative data is also available in Alberta, including the ability to track all physician claims, and subsequent long-term dialysis use for study participants, using methods familiar to study investigators. For secondary analyses to inform policy makers in other countries, we will obtain similar costing information from additional participating countries to evaluate the cost-effectiveness in varying economic contexts and enable the broadest possible generalizability and policy relevance of our analysis.

### Participating centers

Participating institutions will be expected to be familiar with the proper utilization of the ANPPT device or else undergo an in service with a content matter expert on ANPTT device utilization prior to site participation. For both arms of the trial, it will be expected that attending surgeons are involved in either the direct supervision and/or inter-operative participation with either facial closure or temporary abdominal closure in order to be an acceptable participating center. Further criteria required for potential participating centers are presented in Table 4.

**Table 4:** Site requirements for potential participation in the COOL study

Minimal system resources required for site participation in COOL-Lite	
- Designated primary investigator presumably with an academic affiliation willing to take overall medical/ethical/academic responsibility for the conduct of the study	
- Ethical approval—by the appropriate local ethics committee with oversight of the participating institution	
- Site investigators/willing local surgeons with the responsibility of caring for those with SIAS and thus the ability to recruit patients	
- Internet access—either within or closely available to the operating theater to allow online randomization of patients during laparotomy	
- Negative peritoneal pressure therapy (NPPT) dressing availability for those randomized to OPEN	
- Familiarity with the application of the NPPT device or willingness to undergo training and in service on the safe utilization of the NPPT device	
- Study personnel/investigator capable to record and compile case record and submit to the Central Study Registry	
Full system resources required for site participation in COOL-Max	
- Above and also	
- Study personnel capable of obtaining blood/IPF samples	
- Laboratory capability to store frozen blood/IPF fluid till study completion and send to Calgary for analysis	
Full system resources required for site participation in COOL-Mic	
- Medical microbiology laboratory capable of basic microbiology studies	
- Medical records and information processing capable of providing microbiology results for study analysis	
Full system resources required for site participation in COOL-Cells	
- Geographic proximity to Calgary	
- Ability to collect fresh peritoneal fluid and to rapidly ship to the Snyder laboratory for time-of-flight mass spectrometer	
Full system resources required for site participation in COOL-Costs	
- Ability to provide administrative and microcosting data	
- Ability to administrator SF-36 and Euroqol EQ-5D-5L at 90 days and 1 year post enrollment in all survivors	

### Sample size calculations

The COOL trial will overall be powered to detect a significant difference in the primary outcome, 90-day survival. While there is little solid data with which to integrate, the preceding peritoneal VAC study revealed an intention-to-treat 90-day mortality of 21.7% in the ABThera group versus 50.0% in the Barker’s vacuum pack group [HR, 0.32; 95% confidence interval (CI), 0.11–0.93; *P* = 0.04] [60]. This 30% reduction in mortality is likely too dramatic to expect to be practically replicated, and thus, a more conservative effective of 10% reduction in mortality would be appropriate. Thus, given a mortality rate of 33% in the general population of those with severe intra-abdominal sepsis, and considering a power of 80% and an alpha of 0.05, the number needed to recruit in each arm is 275 patients.

### Statistical analyses

The effectiveness of randomization will be displayed through a detailed presentation of patient demographic characteristics as outlined in Table 5. The analysis of the primary outcome, mortality, will be on an intention-to-treat basis related to the allocation of initial intra-operative therapy. There will be a planned subgroup analysis of the actuarial mortality stratifying patients into those with and without the presence of septic shock (defined as Sepsis-3 Consensus Guidelines) during the first 48 h after onset of peritonitis (if known and 24 h before and 24 h after the first laparotomy if not known). Secondary outcomes are described in Table 2. For the comparison of health-care costs, we will use established methods to enable comparisons of mean costs, as these are easily interpretable and relevant to health-care payer. We will include the full cost of the intervention, as well as the hospital costs for the cost categories noted above (for both groups) and will use non-parametric bootstrap estimates to derive 95% confidence interval (95% CI) and mean cost differences between the treatment arms. We will use 1000 bias-corrected bootstrap replications (including sampling with replacement from the original data) to estimate the distribution of a sampling statistic to derive 95% confidence intervals. In sensitivity analyses, we will also use generalized linear models to compare total costs across groups, considering three family distributions (Gaussian, inverse Gaussian, and gamma) and specifying two link functions (identity and log).

**Table 5:** Baseline demographic characteristics of the study patients

Male/female,	
Age, median (IQR^a^), years	
Septic shock^b^	
World Society of Emergency Surgery Sepsis Severity Score^c^	
Calgary PIRO Score^d^	
GCS^e^, median (IQR)	
APACHE II^f^, median (IQR)	
Arterial pH, mean (95% CI)	
Base deficit, median (IQR)	
Lactate, median (IQR)	
INR^g^, median (IQR)	
Temperature, mean (95% CI)	
APACHE-II score^f^, mean ± SDd	
SOFA score^h^, mean ± SDe	
Charlson Comorbidity Index score^i^, median (IQR)	
Worst physiologic measurements prior to randomization, median (IQR)	
Systolic blood pressure, mmHg	
Temperature (injured patients), °C	
Temperature (sepsis patients), °C	
pH	
Lactate, mmol/L	
Base deficit, mmol/L	
INR	
Fluid administration prior to randomization, median (IQR)	
PRBC^j^, units	
FFP^k^, units	
PRBC/FFP ratio	
Crystalloid, L	
Patient location prior to OR admission—no. (%)	
Emergency department	
Hospital ward	
Intensive care unit	
Vasopressors required prior to randomization—no. (%)	
Hours from sepsis diagnosis to laparotomy, median (IQR)	

^a^IQR interquartile range

^b^Septic shock as defined by SESPS-3 guidelines [7]

^c^WSESSS [8]

^d^CPIRO [43]

^e^*GCS* Glasgow Coma Score

^f^Acute Physiology And Chronic Health Evaluation II

^g^*INR* international normalized ratio

^h^*SOFA* Sequential Assessment of Organ Failure [110]

^i^Charlson Comorbidity Index [111]

^j^*PRBC* packed red blood cells

^k^*FFP* fresh frozen plasma

**Fig. 2:**
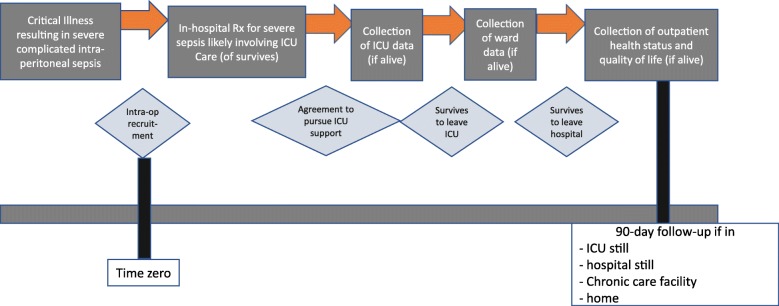
Participant time line for COOL recruitment

There will be a single interim analysis planned after the recruitment of 275 patients, which will analyze the difference in 90-day mortality between allocated therapies. The COOL investigators appreciate the general reluctance to stop randomized trials early due to the benefit and due to the frequent over-estimating of treatment effects [61–63]. Despite this, it is possible that the COOL trial will be great over-powered as although the sample size calculations are based on the best outcome data from randomized trials of NPPT, this is still inferential as there is no previous relevant data with which to accurately guide such calculations. Thus, if a profoundly significant difference is found (*p* < 0.01), the trial will be stopped; otherwise, it will continue to full recruitment (Fig. 2).

### Ethical concerns

The Hippocratic Oath requires physicians to “consider for the benefit of my patients and abstain from whatever is deleterious and mischievous” and to “give no deadly medicine to any one if asked, nor suggest any such counsel”. Thus philosophically, as there is complete clinical equipoise concerning the treatment of SCIAS with or without the OA technique, the COOL investigators feel a moral imperative to provide the best evidence to counsel bedside critical care physicians and surgeons [64]. The COOL trial is currently approved by the Conjoint Research Ethics Board of the University of Calgary (REB-16-1588) to proceed with a delayed consent process given the time-sensitive critical nature of decision making. Research ethics will vary throughout the world, and it is anticipated that various local policies concerning community consent, waiver of consent, or informed consent of significant patient proxies will vary among the local approaches to ensure the COOL trial is performed to the highest ethical standards on a global basis. All participating institutions will thus be required to obtain ethical approval appropriate and applicable to their institutions.

## Discussion

The COOL trial has been designed to answer a critical clinical question that faces clinicians worldwide on a daily basis for which there is great clinical equipoise and potential severe consequences for patients in regard to potential poor outcomes [30, 42]. Thus, this question has been identified as one requiring urgent study by the most contemporary of international reviews [52]. Subsequently, the COOL trial has been endorsed by numerous scientific organizations with vested interest in the best care of the critically ill patient including the Abdominal Compartment Society, the World Society of Emergency Surgery, the Trauma Association of Canada, the Canadian Association of General Surgeons, and the Canadian Hernia Society. The trial design and vision follow directly from the preceding single-center study of differing modalities of APNPPT conducted at the Foothills Medical Centre, which noted a survival advantage associated with a more efficient APNPPT without being able to confirm a biological mechanism of such [41, 60]. When the peritoneal VAC investigators considered following up the pilot study and enrolling more patients in a multi-center fashion, it became apparent that any differing effectiveness of ANPPT techniques was not the most relevant question concerning the OA. With a dramatic evolution in resuscitation practices involving balanced resuscitation practices, more and more trauma patients who previously become so edematous required OA therapy, are no longer being crystalloid over-resuscitated, and can thus be primarily closed [65–67]. This dramatic change in the trauma care paradigm has justified questions regarding the whole premise of Damage Control Surgery for trauma [68] and justifies the randomized control trial of the practice in trauma patients [69].

As over-resuscitation becomes rare, and de-resuscitation becomes a focus [70], it is intuitive that there will be more abdomens in non-trauma intra-abdominal sepsis patients who may be technically closed without inducing intra-abdominal hypertension (IAH). However, although these abdomens *may* be closed, *should* they be closed? As has been recently emphasized, there are profound differences in the basic science of sepsis and traumatic injury [71], with the previously unifying concepts of non-infectious Systemic Inflammatory Response Syndrome (SIRS) being effectively discarded as a clinically helpful construct [7, 72, 73]. The one nebulous, poorly defined “holy-grail” of the optimal management of SCIAS is adequate “source control.” It is suggested that even if an abdomen can be physically closed, there may be an advantage to leaving it open to allow better drainage of intra-peritoneal contamination, a concept that is supported by remarkable animal lab data suggesting the ability of ANPPT to mitigate the elaboration of the inflammatory bio-mediator cascade [39, 40, 74]. Coupled with technical advances in ANPPT dressings that are safer to utilize and that increasingly protect the viscera, this appears an attractive option for the sickest IAS patients.

### The peritoneal cavity as a reservoir for systemic inflammation

There is a complex relationship between pressure, ischemia, and inflammation within the peritoneal cavity [10]. Independently, the damaged gut seems to act as a continued source of inflammation propagating SIRS and potentiating MODS [75–77]. Although extremely complicated, visceral ischemia further characteristically generates multiple immunological mediators with the pro-inflammatory cytokines tumor necrosis factor-alpha (TNF-α), and interleukin 6 (IL-6), as well as inhibitive cytokines such as interleukin 10 (IL-10) [78–81]. Post-operative complications are associated with increasing levels of systemic IL-6 and peritoneal TNF-α [80, 82]. Jansson and colleagues believe that peritoneal cytokines in humans respond more extensively compared to systemic cytokine and that a normal postoperative course is characterized by decreasing levels of peritoneal cytokines based on studies of both elective and emergency surgery [83]. Overall, the peritoneal cytokine response is much higher than the systemic response in peritonitis [81, 84–86]. Hendriks demonstrated that peritoneal cytokine levels (especially IL-6, TNF-α [87], and IL-10) were dramatically different in rats who either survived or succumbed to an intra-peritoneal sepsis model in the 24 h after cytokine determination [84]. Finally, the recent work suggests that blood filters designed to hemofiltrate blood endotoxins and cytokines may improve hemodynamics, organ dysfunction, and even mortality in the critically ill [88–91].

We believe that if it can be done safely, it is logical to attempt to remove intra-peritoneal bio-mediators to potentially ameliorate the local effects and to prevent them being absorbed systematically. Although early uncontrolled work suggested benefit to simple continuous peritoneal lavage after either gross peritoneal contamination in secondary peritonitis or in the setting of necrotizing pancreatitis [92, 93], more structured studies could not confirm such benefits [94–96]. Thereafter, work focused upon using hemofiltration to remove inflammatory mediators from the blood which has been associated with reduced elevations of inflammatory cytokines (as assessed by blood IL-6 levels), early improvements of hemodynamic state, and decreased lactate levels [97–99]. In an attempt to comprehensively increase efficiency, the potential utility of adding extra-corporeal mediator removal through hemofiltration in addition to continuous peritoneal lavage have been entertained and studied in early models [91].

ANNPT therapy may be a more direct and focused solution to this complicated problem and one that will be complementary to the other benefits of OA use in the sickest patients. Whether improved post-operative courses can be obtained through this relatively simpler approach of actively removing peritoneal cytokines with a more efficient and comprehensive VAC therapy in humans is therefore a stated secondary but important objective of the COOL-MAX arm of this trial.

Another potential benefit of ANPTT after severe infection may be the attendant decompression of the abdominal compartment and prevention of even modest degrees of IAH. Patients with intra-abdominal infections are at risk of elevated IAP both as a result of the primary intra-peritoneal disease, as any large fluid resuscitation often required to maintain organ perfusion [100–102]. Recent studies have demonstrated a high prevalence of IAH following aggressive resuscitation of septic patients. Intra-abdominal hypertension is present in as many as 80% of septic medical and surgical ICU patients [103, 104]. Reintam also reported that septic patients with IAH had a 50% rate of mortality compared to 19% without IAH, making IAH a significant marker for an increased risk of death [105]. Within our own institution, rates of IAH were over 87% of septic ICU patients, and further 61% of these patients had severe IAH at levels commensurate with ACS, despite the fact that IAP was only measured in 10% of the patients in whom guidelines recommend monitoring [106]. Although direct translation to humans is uncertain, even modest degrees of IAH (often clinically ignored) have been found to have profound far-reaching effects on propagating multiple organ failure in animals with ischemia/intra-peritoneal infections [107–109].

The investigators and the scientific community have extensively reviewed and critiqued the results of the preceding peritoneal VAC trial [90]. Methodologic concerns with the peritoneal VAC trial were that it enrolled quite heterogeneous patients with a wide range of ages and included traumatized patients with an exactly known time of injury and severe IAS patients in whom the timing of onset of severe disease was inexactly known. Thus, the COOL trial will focus on a less heterogeneous group of patients with intra-operatively confirmed SIAS in order to increase the signal to noise ratio. The Steering Group considers conducting the COOL trial to be a practical undertaking. With a projected total necessary recruitment of 550 patients and with an average institutional estimate of 10 such patients recruited per year, this projects to 28 actively engaged centers globally for 2 years. The initial expressed interest far exceeds this number of centers, and it is likely that many will be able to recruit many more than 10 per year. For example, at the Pilot Center, Foothills Hospital in Calgary, Canada, the group was extremely supportive with the similar recruitment process of the peritoneal VAC trial, in which out of 63 potentially eligible patients, 45 (71%) were recruited over 15 months [27], with reasons for non-recruitment including gynecological procedures and rescue laparotomies outside of a regular operating room.

## Conclusions

The COOL trial will be powered on demonstrating a mortality difference in this highly lethal and morbid condition to ensure critically ill patients are receiving the best care possible and not being harmed by inappropriate therapies based on opinion only. As this will constitute a major international collaborative, undertaking a number of secondary outcomes will be conducted that are expected to add to the basic and translational science of SCIAS.

## Abbreviations

ANPPT: Active negative pressure peritoneal therapy
APACHE: Acute Physiology and Chronic Health Evaluation
APC: Activated protein C
ARDS: Acute Respiratory Distress Syndrome
AUC: Area under the curve
C3a: Complement factor 3 activated
C5a: Complement factor 5 activated
COOL: Closed Or Open after Laparotomy trial (https://clinicaltrials.gov/ct2/show/NCT03163095)
CPIRO: Calgary Predisposition Infection Response Organ Dysfunction
EAF: Enteroatmospheric fistula
LOD: Laparotomy on demand
MODS: Multiple Organ Dysfunction Score
mtDNA: Mitochondrial DNA
NPPT: Negative pressure peritoneal therapy
OA: Open abdomen
PRL: Planned re-laparotomy
qSOFA: Quick SOFA score
RCT: Randomized controlled trial
SCIAS: Severe complicated intra-abdominal sepsis
SIRS: Systemic Inflammatory Response Syndrome
SOFA: Sequential Organ Failure Assessment
WSESSSS: World Society of Emergency Surgery Sepsis Severity Score
